# Data-analysis strategies for image-based cell profiling

**DOI:** 10.1038/nmeth.4397

**Published:** 2017-09-01

**Authors:** Juan C Caicedo, Sam Cooper, Florian Heigwer, Scott Warchal, Peng Qiu, Csaba Molnar, Aliaksei S Vasilevich, Joseph D Barry, Harmanjit Singh Bansal, Oren Kraus, Mathias Wawer, Lassi Paavolainen, Markus D Herrmann, Mohammad Rohban, Jane Hung, Holger Hennig, John Concannon, Ian Smith, Paul A Clemons, Shantanu Singh, Paul Rees, Peter Horvath, Roger G Linington, Anne E Carpenter

**Affiliations:** 1 Imaging Platform, Broad Institute of Harvard and MIT, Cambridge, Massachusetts USA; 2 Imperial College London, London, UK; 3 German Cancer Research Center and Heidelberg University, Heidelberg, Germany; 4 Institute of Genetics & Molecular Medicine, University of Edinburgh, Edinburgh, UK; 5 Department of Biomedical Engineering, Georgia Institute of Technology and Emory University, Atlanta, Georgia USA; 6 Synthetic and System Biology Unit, Hungarian Academy of Sciences, Szeged, Hungary; 7 Laboratory for Cell Biology–Inspired Tissue Engineering, MERLN Institute, Maastricht University, Maastricht, The Netherlands; 8 Department of Biostatistics and Computational Biology, Dana-Farber Cancer Institute, Boston, Massachusetts USA; 9 National Centre for Biological Sciences, Bangalore, India; 10 Electrical and Computer Engineering, University of Toronto, Toronto, Ontario Canada; 11 Chemical Biology and Therapeutics Science Program, Broad Institute of MIT and Harvard, Cambridge, Massachusetts USA; 12 Institute for Molecular Medicine Finland (FIMM), University of Helsinki, Helsinki, Finland; 13 Institute of Molecular Life Sciences, University of Zurich, Zurich, Switzerland; 14 Department of Chemical Engineering, Massachusetts Institute of Technology, Cambridge, Massachusetts USA; 15 Department of Systems Biology & Bioinformatics, University of Rostock, Rostock, Germany; 16 Department of Chemical Biology and Therapeutics, Novartis Institutes for Biomedical Research, Cambridge, Massachusetts USA; 17 Connectivity Map Project, Broad Institute of Harvard and MIT, Cambridge, Massachusetts USA; 18 College of Engineering, Swansea University, Swansea, UK; 19 Department of Chemistry, Simon Fraser University, Burnaby, British Columbia Canada

**Keywords:** Image processing, Machine learning

## Abstract

Image-based cell profiling is a high-throughput strategy for the quantification of phenotypic differences among a variety of cell populations. It paves the way to studying biological systems on a large scale by using chemical and genetic perturbations. The general workflow for this technology involves image acquisition with high-throughput microscopy systems and subsequent image processing and analysis. Here, we introduce the steps required to create high-quality image-based (i.e., morphological) profiles from a collection of microscopy images. We recommend techniques that have proven useful in each stage of the data analysis process, on the basis of the experience of 20 laboratories worldwide that are refining their image-based cell-profiling methodologies in pursuit of biological discovery. The recommended techniques cover alternatives that may suit various biological goals, experimental designs, and laboratories' preferences.

## Abstract (web summary)

This Review covers the steps required to create high-quality image-based profiles from high-throughput microscopy images.

## Main

Image analysis is heavily used to quantify phenotypes of interest to biologists, especially in high-throughput experiments^1,2,3^. Recent advances in automated microscopy and image analysis allow many treatment conditions to be tested in a single day, thus enabling the systematic evaluation of particular morphologies of cells. A further revolution is currently underway: images are also being used as unbiased sources of quantitative information about cell state in an approach known as image-based profiling or morphological profiling^4^. Herein, the term morphology will be used to refer to the full spectrum of biological phenotypes that can be observed and distinguished in images, including not only metrics of shape but also intensities, staining patterns, and spatial relationships (described in 'Feature extraction').

In image-based cell profiling, hundreds of morphological features are measured from a population of cells treated with either chemical or biological perturbagens. The effects of the treatment are quantified by measuring changes in those features in treated versus untreated control cells^5^. By describing a population of cells as a rich collection of measurements, termed the 'morphological profile', various treatment conditions can be compared to identify biologically relevant similarities for clustering samples or identifying matches or anticorrelations. This profiling strategy contrasts with image-based screening, which also involves large-scale imaging experiments but has a goal of measuring only specific predefined phenotypes and identifying outliers.

Similarly to other profiling methods that involve hundreds of measurements or more from each sample^6,7^, the applications of image-based cell profiling are diverse and powerful. As reviewed recently^8,9^, these applications include identifying disease-specific phenotypes, gene and allele functions, and targets or mechanisms of action of drugs.

**Figure 1:**
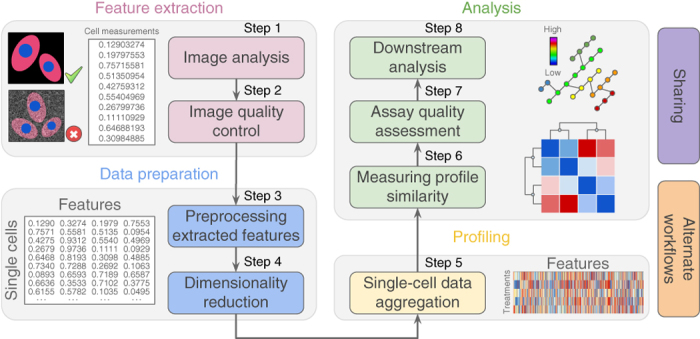
Representative workflow for image-based cell profiling. Eight main steps transform images into quantitative information to support experimental conclusions.

However, the field is currently a wild frontier, including novel methods that have been proposed but not yet compared, and few methods have been used outside the laboratories in which they were developed. The scientific community would greatly benefit from sharing methods and software code at this early stage, to enable more rapid convergence on the best practices for the many steps in a typical profiling workflow (Fig. 1).

Here, we document the options at each step in the computational workflow for image-based profiling. We divide the workflow into eight main steps (Fig. 1). For each step, we describe the process, its importance, and its applicability to different experimental types and scales. We present previously published methods relevant to each step, provide guidance regarding the theoretical pros and cons for each alternative option, and refer to any prior published comparisons of methods. We do not cover the upstream steps (sample preparation and image-acquisition recommendations)^1,2^ or computational practicalities such as the necessary information-technology infrastructure to store and process images or data. The workflow's starting point is a large set of images. The assays can be specifically designed for profiling, such as Cell Painting^10,11^, but any image-based assays can be used, including a panel of multiple parallel image-based assays^12^, or time-lapse microscopy for analyzing dynamics^13^ or even whole organisms^14^.

This paper is the result of a 'hackathon', in which the authors met to discuss and share their expertise in morphological profiling. Hands-on data-analysis challenges and the accompanying discussions helped to identify the best practices in the field and to contribute algorithms to a shared code base.

We hope to provide a valuable foundation and framework for future efforts and to lower the barrier to entry for research groups that are new to image-based profiling. The detailed workflows used by each individual laboratory contributing to this article can be found online (https://github.com/shntnu/cytomining-hackathon-wiki/wiki/).

### Step 1: image analysis

Image analysis transforms digital images into measurements that describe the state of every single cell in an experiment. This process makes use of various algorithms to compute measurements (often called features) that can be organized in a matrix in which the rows are cells in the experiment, and the columns are extracted features.

**Field-of-view illumination correction.** Every image acquired by a microscope exhibits inhomogeneous illumination mainly because a nonuniform light source or optical path often yields shading around edges. This effect is often underestimated; however, intensities usually vary by 10–30%, thus corrupting accurate segmentation and intensity measurements^15^. Illumination correction is a process to recover the true image from a distorted one. There are three main approaches to illumination correction:

*Prospective methods*. These methods build correction functions from reference images, such as dark and bright images with no sample in the foreground. The approach requires careful calibration at the time of acquisition and relies on assumptions that are often inappropriate, thus yielding an incomplete correction in practice^16^.

*Retrospective single-image methods*. These methods calculate the correction model for each image individually^17,18,19^. However, the result can change from image to image and thus may alter the relative intensity.

*Retrospective multi-image methods*. These methods build the correction function by using the images acquired in the experiment. These methods are often based on smoothing^16^, surface fitting^20^, or energy-minimization models^15^.

Illumination correction is an important step for high-throughput quantitative profiling; the strategy of choice in most of our laboratories is a retrospective multi-image correction function. This procedure produces more robust results, particularly when separate functions are calculated for each batch of images (often with a different function for each plate and always with a different function for different imaging sessions or instruments). We recommend use of prospective and single-image methods for only qualitative experiments.

**Figure 2:**
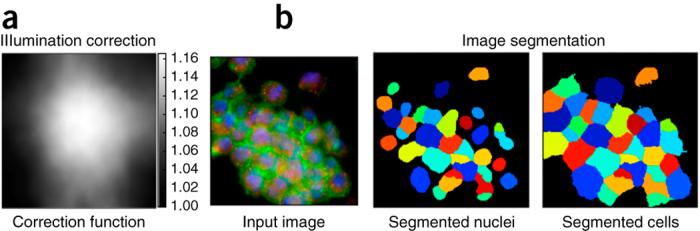
Methods used for image analysis. (**a**) Illumination-correction function estimated with a retrospective multi-image method. Pixels in the center of the field of view are systematically brighter than pixels in the edges. (**b**) Image segmentation aims to classify pixels as either foreground or background, i.e. as being part of an object or not. Here, regions have been segmented with the model-based approach.

**Segmentation.** Typically, each cell in the image is identified and measured individually; that is, its constituent pixels are grouped to distinguish the cell from other cells and from the background. This process is called 'segmentation' (Fig. 2), and there are two main approaches:

*Model based*. The experimentalist chooses an appropriate algorithm and manually optimizes parameters on the basis of visual inspection of segmentation results. A common procedure is first to identify nuclei, as can often be done easily, and then to use the results as seeds for the identification of the cell outline. A priori knowledge (i.e., a 'model') is needed, such as the objects' expected size and shape^21^. Model-based approaches typically involve histogram-based methods, such as thresholding, edge detection, and watershed transformation^22^.

*Machine learning.* A classifier is trained to find the optimal segmentation solution by providing it with ground-truth data and manually indicating which pixels of an image belong to different classes of objects^23^. This approach typically involves applying various transformations to the image to capture different patterns in the local pixel neighborhood. Segmentation is ultimately achieved by applying the trained model to new images to classify pixels accordingly.

Both approaches are used in profiling experiments. The model-based approach is most common (for example, in CellProfiler^24^); it performs well for fluorescence microscopy images of cultured cells^22^. However, it requires manual parameter adjustment for each new experimental setup. Machine-learning-based segmentation (for example, in Ilastik^23^) can perform better on difficult segmentation tasks, such as highly variable cell types or tissues. It does not require as much computational expertise, but it does require manual labeling of training pixels for each experimental setup and sometimes even for each batch of images. The creation of ground-truth data in the process of labeling allows for quantitative performance assessment.

**Feature extraction.** The phenotypic characteristics of each cell are measured in a step called feature extraction, which provides the raw data for profiling. The major types of features are:

*Shape features*. These features are computed on the boundaries of nuclei, cells, or other segmented compartments. These include standard size and shape metrics such as perimeter, area, and roundness^25,26^.

*Intensity-based features*. These features are computed from the actual intensity values in each channel of the image on a single-cell basis, within each compartment (nucleus, cell, or other segmented compartments). These metrics include simple statistics (for example, mean intensity, and maximum intensity).

*Texture features*. These features quantify the regularity of intensities in images, and periodic changes can be detected by using mathematical functions such as cosines and correlation matrices. These features have been extensively used for single-cell analysis^27,28,29,30^.

*Microenvironment and context features*. These features include counts and spatial relationships among cells in the field of view (on the basis of the number of and distance to cells in a neighborhood) as well as its position relative to a cell colony^31,32,33^. Segmented regions are not limited to nuclei, and cells and may also include subcellular structures that can be quantified as measurements (for example, speckles within a nucleus or distances between the nucleus and individual cytoplasmic vesicles).

Whereas screening experiments typically measure one or two features of interest to quantify specific effects^34^, cell profiling involves computing as many features as possible to select robust, concise, and biologically meaningful features to increase the chances of detecting changes in the molecular states of cells. The most common practice is to measure hundreds or even thousands of features of many varieties; the details are typically described in the software's documentation^24,35,36^.

### Step 2: image quality control

It is largely impossible to manually verify image quality in high-throughput experiments, so automated methods are needed to objectively flag or remove images and cells that are affected by artifacts. These methods seek to decrease the risk of contaminating the data with incorrect values.

### Field-of-view quality control.

Images can be corrupted by artifacts such as blurring (for example, improper autofocusing) or saturated pixels (for example, debris or aggregations that are inappropriately bright). Typically, statistical measures of image intensity are used for quality control.

Metrics can be computed to detect blurring, including the ratio of the mean and the s.d. of each image's pixel intensities, the normalized measure of the intensity variance^37^, and the image correlation across subregions of the image^38^. The log–log slope of the power spectrum of pixel intensities is another effective option, because the high-frequency components of an image are lost as it becomes more blurred^39^; this procedure has been found to be the most effective in a recent comparison for high-throughput microscopy^40^. For detecting saturation artifacts, the percentage of saturated pixels has been found to be the best among all tested metrics.

We recommend computing various measures that represent a variety of artifacts that might occur in an experiment to increase the chance of artifact identification. Then, with data-analysis tools, these measurements can be reviewed to identify acceptable quality-control thresholds for each measure^40^. It is also possible to use supervised machine-learning algorithms to identify problematic images^41,42^, but these algorithms require example annotations and classifier training and validation, and thus may require more effort and introduce a risk of overfitting.

**Cell-level quality control.** Outlier cells may exhibit highly unusual phenotypes but may also result from errors in sample preparation, imaging, image processing, or image segmentation. Errors include incorrectly segmented cells, partly visible cells at image edges, out-of-focus cells, and staining artifacts. Although errors are best decreased through careful techniques and protocols, there are several strategies for detecting outlier cells:

*Model-free outlier detection*. This strategy includes methods to define normal limits by using statistics. Data points represented with a single variable (for example, distance values or single features) can be analyzed with univariate statistical tools, including the 3- or 5-s.d. rules, Winsorizing, and the adjusted box-plot rule^43^. Robust statistics based on estimators such as the median and the median absolute deviation^44^ can also be used and extended to multivariate situations^45^. Additional multivariate methods include principal component analysis (PCA) and Mahalanobis-based outlier detection^46^.

*Model-based outlier detection*. This strategy involves training a model of normal samples to aid in detecting outlier cells^47^. For instance, if a linear regression among features is suitable, outliers can be detected as data points with a large residual that does not follow the general trend^48^. Alternately, a supervised-machine-learning classifier can be trained by providing examples of outliers^49,50,51^.

**Figure 3:**
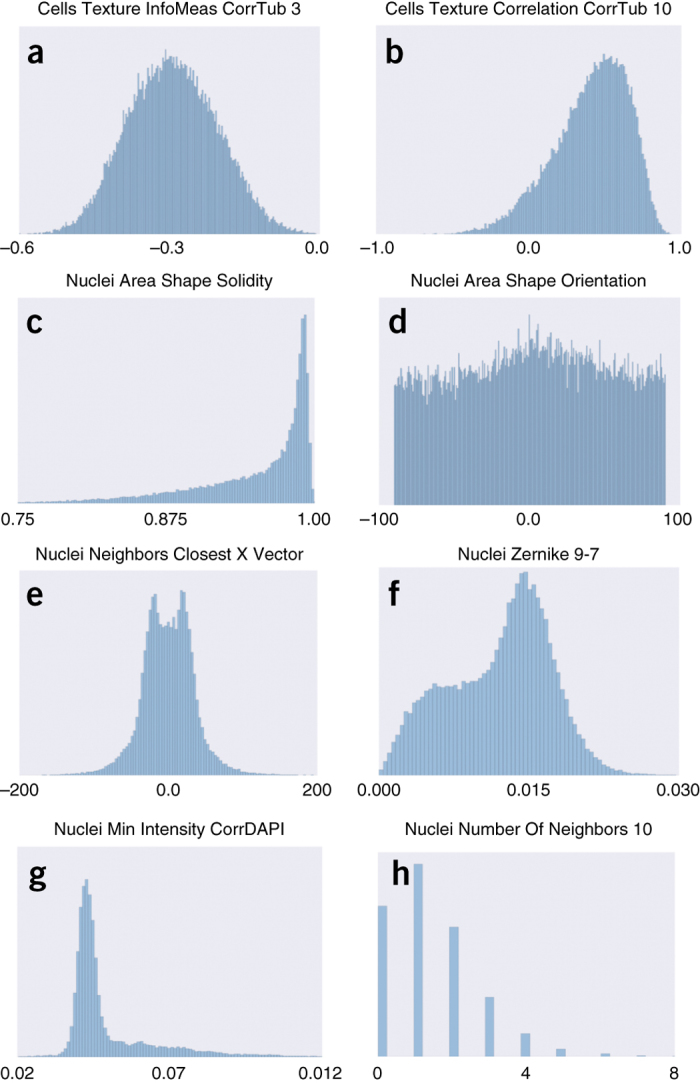
Diversity of feature distributions in morphological profiling. (**a**–**h**) Morphological features display various types of distributions, including normal (**a**), skewed (**b**,**c**), uniform (**d**), multimodal (**e**–**g**), and even discrete distributions (**h**). The ranges in which features are represented also vary considerably. These histograms were obtained with feature values from a sample of 10,000 cells in the BBBC021 data set^108^. The names of features correspond to conventions used in the CellProfiler software. The *x* axes show feature values (in different units), and the *y* axes show frequencies (cell counts).

After they are detected, outlier cells can be removed, or when the number of outliers in the sample is too high, the entire sample can be examined manually or omitted from analysis^47,52^. Importantly, cell-outlier detection should be performed at the whole-population level; that is, it should not be separately configured per well, per replicate, or per plate. Extreme caution is recommended, to avoid removing data points that represent cells and samples with interesting phenotypes^53,54^. Samples can be composed of various subpopulations of cells, and outlier-detection methods may incorrectly assume normality or homogenous populations (Fig. 3). For this reason, most laboratories skip outlier detection at the level of individual cells, other than to check for segmentation problems.

### Step 3: preprocessing extracted features

Preparing extracted cell features for further analysis is a delicate step that can enhance the observation of useful patterns or can corrupt the information and lead to incorrect conclusions.

**Missing values.** Feature-extraction software may yield non-finite symbols (such as NaN and INF) representing incomputable values. In general, use of these symbols is preferred to assigning a numerical value that could be interpreted as having a phenotypic meaning. The presence of non-finite symbols poses challenges to applying statistics or machine-learning algorithms. There are three alternate solutions for handling missing values:

*Removing cells*. If a small proportion of cells have missing values, excluding them can be considered. However, those cells may indicate a valid and relevant phenotype, a possibility that should be assessed carefully (described in 'Cell-level quality control').

*Removing features*. If a large proportion of cells have a missing value for a particular feature, they might be removed on the grounds that the feature is insufficiently informative. Again, this removal should be assessed carefully for its effect on unexpected cell phenotypes.

*Applying imputation*. If the proportion of cells with missing values for certain features is relatively small, several statistical rules may be applied to complete these values. The use of zeros or the mean value is common in general statistical analysis but should not be the default option for single-cell profiling. If too many values are artificially added to the data matrix, the downstream analysis may be affected or biased by false data.

Deciding how to proceed with missing values is primarily dependent on experimental evaluations and empirical observations. Removing cells or features is more common than applying imputation. However, there is no single rule that applies in all cases, and the best practice is to collect convincing evidence supporting these decisions, especially with the use of quality measures and replicate analysis (described in 'Downstream analysis').

**Plate-layout-effect correction.** High-throughput assays use multiwell plates, which are subject to edge effects and gradient artifacts. Concerns regarding spatial effects across each plate are not unique to imaging and have been widely discussed in both the microarray-normalization and high-throughput-screening literature^44,55,56,57,58^. They can be decreased to some degree at the sample-preparation step^59^.

We recommend checking for plate effects to determine whether any artifacts are present within plates or across multiple batches. The simplest method is a visual check, through plotting a measured variable (often cell count or cell area) as a heat map in the same spatial format as the plate; this procedure allows for easy identification of row and column effects as well as drift across multiple plates.

We recommend using a two-way median polish to correct for positional effects. This procedure involves iterative median smoothing of rows and columns to remove positional effects, then dividing each well value by the plate median absolute deviation to generate a *B* score^60^. However, this procedure cannot be used on nonrandom plate layouts such as compound titration series or controls placed along an entire row or column^54^. Other approaches include 2D polynomial regression and running averages, both of which correct spatial biases by using local smoothing^61^. Notably, image-based profiling is often sufficiently sensitive to distinguish among different well positions containing the same sample. Thus, to mitigate these positional effects, samples should be placed in random locations with respect to the plate layout. However, because such scrambling of positions is rarely practical, researchers must take special care to interpret results carefully and to consider the effects that plate-layout effects might have on the biological conclusions.

**Figure 4:**
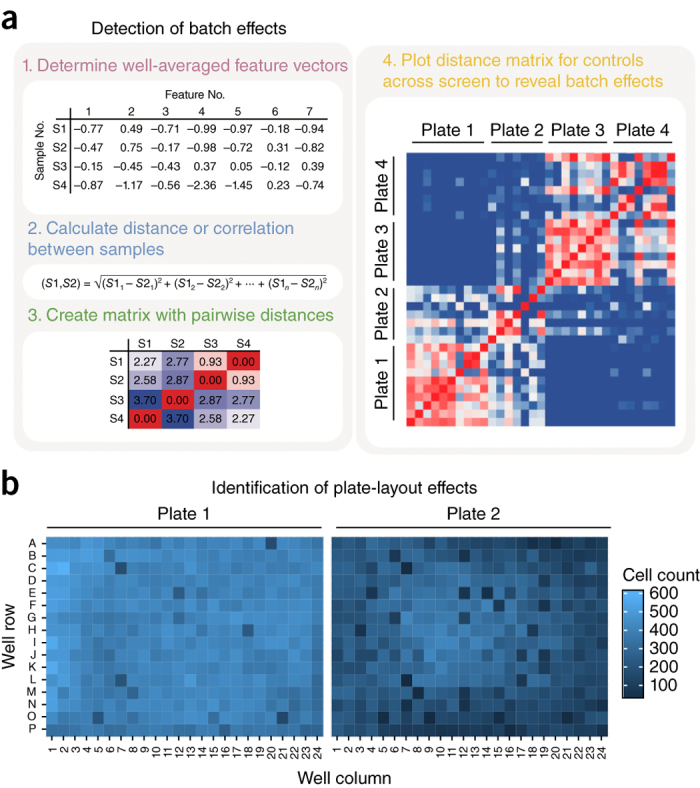
Example diagnostic plots for detecting batch effects and plate-layout effects. (**a**) Process of detecting batch effects. The largest matrix on the right shows how plates 1 and 2 are more correlated to each other than to plates 3 and 4, and vice versa. This pattern suggests that plates 1 and 2, as well as 3 and 4, were prepared in batches that have noticeable differences in their experimental conditions. (**b**) Two plate layouts illustrating the cell count in each well. The visualization allows for identification of plate-layout effects, such as unfavorable edge conditions. Plate 1 shows that cells can grow normally in any well, whereas plate 2 shows markedly lower cell counts at the edges, thus indicating the presence of experimental artifacts.

**Batch-effect correction.** Batch effects are subgroups of measurements that result from undesired technical variation (for example, changes in laboratory conditions, sample manipulation, or instrument calibration) rather than constituting a meaningful biological signal (Fig. 4). Batch effects pose a major challenge to high-throughput methodologies, and correction is an important preliminary step; if undetected, batch effects can lead to misinterpretation and false conclusions^62^.

We recommend identifying batch effects by inspecting correlations among profiles (described in 'Single-cell data aggregation'). Specifically, by plotting heat maps of the correlation between all pairs of wells within an experiment, sorted by experimental repeat, batch effects can be identified as patterns of high correlation corresponding to technical artifacts (Fig. 4a). As a quantitative check, within-plate correlations should be in the same range as across-plate correlations.

When correction is needed, standardization and quantile normalization, as discussed in 'Feature transformation and normalization', can be applied within plates rather than to the entire screen^63^. This procedure should be performed only if samples are relatively randomly distributed across plates. Canonical correlation analysis can also be used to transform data to maximize the similarity between technical replicates across experiments^64,65^. Nonetheless, care should be taken to ensure that batch effects have been correctly decreased without false amplification of other sources of noise.

**Feature transformation and normalization.** Morphological profiles include features that display varying shapes of statistical distributions^66^. It is therefore essential to transform feature values with simple mathematical operations, such that the values are approximately normally distributed and mean centered and have comparable s.d. Normal distributions make it easier to work with numeric values from a mathematical, statistical, and computational point of view. We highlight three key steps in this process:

*Distribution testing*. The need for transforming feature values can be evaluated for each feature on the basis of diagnostic measures and plots (Fig. 3). Graphical methods such as histograms, cumulative distribution curves, and quantile–quantile plots allow for visual identification of features that deviate from symmetric distributions. Analytical tests can also be used, including the Kolmogorov–Smirnov (KS) test and the Kullback–Leibler divergence, both of which aim to compute ratios of deviation from normality.

*Logarithmic transformations*. These transformations are often used to obtain approximate normal distributions for features that have highly skewed values or require range correction^67,68^. Transformations include the generalized logarithmic function^68^ and other adaptations that use shrinkage terms to avoid problems with nonpositive and near-zero feature values^69,70^, as well as the Box–Cox transformation^67^.

*Relative normalization*. This procedure consists of computing statistics (for example, median and median absolute deviation) in one population of samples, and then centering and scaling the rest with respect to that population. Ideally, features are normalized across an entire screen in which batch effects are absent; however, normalization within plates is generally performed to correct for batch effects (described in 'Batch-effect correction'). When choosing the normalizing population, we suggest the use of control samples (assuming that they are present in sufficient quantity), because the presence of dramatic phenotypes may confound results. This procedure is good practice regardless of the normalization being performed within plates or across the screen. Alternately, all samples on a plate can be used as the normalizing population when negative controls are unavailable, too few, or unsuitable for some reason, and when samples on each plate are expected to not be enriched in dramatic phenotypes.

We recommend applying normalization across all features. Normalization can be applied even if features are not transformed, and it is preferable to remove biases while simultaneously fixing range issues. *z*-score normalization is the most commonly used procedure in our laboratories. Normalization also aligns the range of different features, thus decreasing the effects of unbalanced scales when computing similarities (described in 'Measuring profile similarity') or applying analysis algorithms (described in 'Downstream analysis'). It is advisable to compare several transformation and normalization methods, because their performance can vary significantly among assays^71^.

### Step 4: dimensionality reduction

At this point in the workflow, it can be useful to ask which of the measured features provide the most value in answering the biological question being studied.

Dimensionality reduction aims to filter less informative features and/or merge related features in the morphological profiles, given that morphological features calculated for profiling are often relatively redundant. The resulting compact representation is computationally more tractable, and it additionally avoids overrepresentation of similar features, that is, having a subgroup of features that measure similar or redundant properties of cells. Redundant features can diminish the signals of other more complementary features that are underrepresented, thus confounding downstream analysis.

**Feature selection.** Feature selection reduces dimensionality by discarding individual features while leaving the remainder in their original format (and thus retaining their interpretability). Options include:

*Finding correlated features*. One feature is selected from a subgroup that is known to be correlated. For instance, some texture features are highly correlated; thus, not all of them are needed, because they may represent the same underlying biological property. The feature–feature correlation matrix is computed, and pairs with a correlation exceeding a given threshold are identified iteratively. At each step, the feature with the largest mean absolute correlation with the rest of the features is removed.

*Filtering on the basis of replicate correlation*. Features that provide the highest additional information content^69,70^ on the basis of replicate correlation are iteratively selected as follows. An initial set of features is selected, and each of the remaining features is regressed on the selected set. The resulting residual data vector represents the additional information not already present in the selected features. The correlation of this residual vector across replicates is used to quantify information content. As a separate step, features with low replicate correlation are often excluded from analysis because they are too noisy^69,72^.

*Minimum redundancy–maximum relevance*. A subset of features can have high replicate correlation without contributing substantially new information. To prevent selecting redundant features, minimum redundancy–maximum relevance^73^ adds a constraint based on mutual information to the selection algorithm. The resulting selected features have high replicate correlation while preserving a diverse set of measurements^74^.

*Support-vector-machine-based recursive-feature elimination*. A support vector machine is trained to implicitly weigh useful features in a classification task. Then, the features with the lowest weight are iteratively removed until the accuracy of the classification task begins to decline^75^. In profiling applications, it may be desirable to select the features that best separate the treatments from the negative controls^76,77^; the selected features would then be those that maximally differentiate phenotypes.

No previous studies have compared these options. Most groups use the filter method based on replicate correlation^69,70,72^, and some add more powerful algorithms despite the computational cost. A combination of methods could be used, especially in tandem with the replicate-correlation strategy. There are other methodologies that may be useful, such as rescaling features in correlated groups such that their sum is one or selecting the features that contribute to most of the variance in the first two principal components.

**Linear transformation.** Methods of linear transformation seek lower-dimensional subspaces of higher-dimensional data that maintain information content. Linear transformation can be performed on single-cell profiles and aggregated sample-level profiles. Unlike feature selection, transformations can combine individual features, thus making the resulting features more powerful and information rich but potentially impeding their interpretability. Linear transformation across all samples in the experiment is often needed for downstream analysis, to avoid overrepresentation of related features. Options used in morphological profiling are:

*PCA*. This procedure maximizes variance in successive orthogonal dimensions. PCA has been shown to outperform other dimensionality-reduction methods, such as random-forest selection for discriminating small-molecule-inhibitor effects^78^, and independent component analysis and statistical image moments (Zernike/Fourier) for separating cell lines and preserving cell morphology after reconstruction from a lower-dimensional space^79^.

*Factor analysis and linear discriminant analysis*. Factor analysis, which is closely related to PCA, finds nonorthogonal combinations of features representing frequent patterns in the data^80^. Linear discriminant analysis finds a projection that maximizes the separation between positive and negative controls^81^. Both procedures have been successfully used in morphological profiling.

Among our laboratories, and in data science more generally, PCA is the most commonly used choice. Its simplicity and ability to retain a large amount of information in fewer dimensions probably explains its popularity. One comparative analysis using image-based profiling data has shown that factor analysis, compared with some alternate transformations, can identify a compact set of dimensions and improve downstream analysis results^77^.

### Step 5: single-cell data aggregation

**Figure 5:**
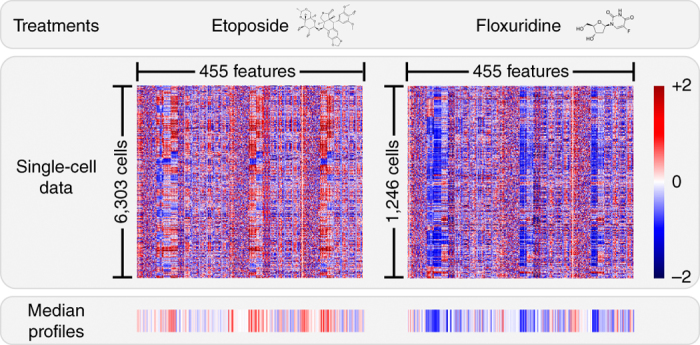
Single-cell data aggregation. The feature matrices of two treatments show the measurements of their cell populations in the experiment. These measurements have been collapsed into median profiles that show very distinct signatures corresponding to two selected compounds: etoposide and floxuridine.

Profiles are data representations that describe the morphological state of an individual cell or a population of cells. Population-level (also called image-level or well-level) representations are obtained by aggregating the measurements of single cells into a single vector to summarize the typical features of the population, so that populations can be compared (Fig. 5).

**Simple aggregations.** There are three simple and commonly used strategies for creating aggregated population-level profiles from all individual cell profiles in the sample:

*Mean profile*. Assuming a normal distribution of features, a profile built from the means of each feature for all cells in the population can provide a useful summary. This method has been used for compound classification^77,82^. The profile length is sometimes doubled by also computing the s.d. of each feature.

*Median profile*. Taking the median for each feature over all the cells in a sample (and optionally the median absolute deviation) can be more robust to non-normal distributions and can mitigate the effects of outliers. If outliers are artifacts or errors, this procedure is useful, but the median may misrepresent populations with rare phenotypes by considering them as undesired outliers.

*KS profile*. This profile compares the probability distribution of a feature in a sample with respect to negative controls by using the KS nonparametric statistical test^83^. The resulting profile is the collection of KS statistics for the features, which reveal how different the sample is with respect to the control.

There are other tests that may perform well but have not been evaluated for morphological profiling. Such tests include the Anderson–Darling statistic and the Mann–Whitney *U* test. Other aggregation strategies can be designed by using bootstrap estimators previously used for phenotype classification^84^.

The median profile has been found to have better performance than other profiling strategies in two different studies^16,77^ and is the preferred choice in most of our laboratories. One choice that varies among groups is whether to construct profiles at the level of images, fields of view, wells, or replicates. One could, for example, calculate a mean profile across all cells in a given replicate (regardless of the image or well) or instead calculate means for each image individually and then calculate means across images to create the replicate-level profile.

**Subpopulation identification and aggregation.** In most image-based cell-profiling workflows, it is implicitly assumed that ensemble averages of single-cell measurements reflect the dominant biological mechanism influenced by the treatment condition. However, subpopulations of cells are known to exhibit different phenotypes even within the same well^85,86^. Classifying populations of single cells on the basis of their shape^87,88,89,90^, cell-cycle phase^13,88,91^, or signaling state^92^ can aid in interpretation and visualization of cell-profiling data^93^. Cellular heterogeneity poses practical challenges for effective measurement methods that account for this variability.

Making use of subpopulations usually involves three key steps:

*Subpopulation identification*. Cells are clustered according to their morphological phenotypes, by using single-cell profiles (from controls or from the whole experiment). Clustering can be supervised, wherein reference phenotypes are selected^94,95,96^, or unsupervised, as in *k*-means clustering^90,97^ and Gaussian mixture model fitting^92^.

*Classification*. Single-cell data points from all treatment conditions are then assigned to one of the subpopulations identified in the previous step. This assignment can be done by using a feature-evaluation rule, such as proximity, similarity, or feature weighting. This step is necessary because subpopulation identification is typically performed only on a subset of cells.

*Aggregation*. For each treatment condition, vectors are calculated and yield the number (or fraction) of cells within each subpopulation. Thus, the dimensionality of these vectors is the number of identified subpopulations.

An unproven hypothesis in the field is that profiles based on identification of phenotypically coherent subpopulations of cells should improve the accuracy of profiling, given the prevalence of heterogeneity and the existence of small subpopulations that might be ignored in mean or median profiling. In fact, to date, subpopulation-based profiling has not improved separation of treatment conditions^77,98^. Nonetheless, defining subpopulations can assist in inferring biological meaning, by identifying over- and underrepresented subpopulations of cells under a given treatment condition^99^ and by improving understanding of the dynamics of how cells transition between different phenotypes^98,100^.

### Step 6: measuring profile similarity

A key component of downstream analysis is the definition of a metric to compare treatments or experimental conditions. Similarity metrics reveal connections among morphological profiles.

**Figure 6:**
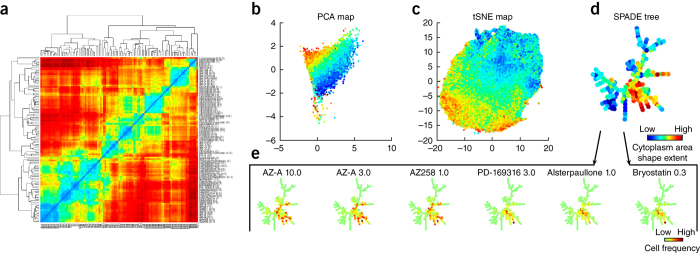
Visualizations for downstream analysis. The source data are from 148,649 cells from the BBBC021 data set^108^. (**a**) A heat map of the distance matrix shows the correlations between all pairs of samples, by using sample-level data (described in 'Measuring profile similarity'). (**b**–**d**) The cellular heterogeneity landscape can be visualized from single-cell data by using PCA (**b**), tSNE scatter plots (**c**) or a SPADE tree (**d**). In these examples, single-cell data points are colored according to a single-cell shape feature 'cytoplasm area shape extent' (red, high; blue, low). (**e**) A separate visualization for each treatment can assist in interpreting phenotypic changes induced by sample treatments. A constant SPADE tree is shown, and treatment-induced shifts in the number of cells in each 'node' of the tree are shown by the color scale depicted. The first three treatments are known to have a similar functional effect (Aurora kinase inhibition), and they exhibit similar cell distributions on the SPADE tree. The remaining three treatments are known to induce protein degradation, inducing cell distributions that differ from the first three.

**Similarity-metric calculation.** With a suitable metric, the similarities among a collection of treatment conditions can facilitate downstream analysis and allow for direct visualization of data structure, for example in distance heat maps (Fig. 6a). Image-based cell-profiling studies use three types of metrics:

*Distance measures*. These measures involve calculating how far apart two points are in the high-dimensional feature space. Those used in morphological profiling include Euclidean^72,83^, Mahalanobis^101^, and Manhattan distances. Distance measures are very useful to quantify the difference in magnitude between profiles, because they aggregate the lengths of feature variations regardless of directionality. This procedure is useful to compute estimates of phenotypic strength of treatments with respect to controls.

*Similarity measures*. These measures involve computing a statistical estimate of the likelihood of a relation between two profiles. Statistics used in morphological profiling include Pearson's correlation^102^, Spearman's rank correlation^103^, Kendall's rank correlation^78^, and cosine similarity^77^. Similarity measures quantify the proximity between profiles, because they detect deviations from one sample to another regardless of the absolute magnitude. This procedure is useful in finding relations and groups of samples that share common properties.

*Learned similarity measures*. These measures involve training machine-learning models that weight features differently according to prior knowledge about samples. The model can be a classifier that systematically identifies differences between two samples by using cross-validation^104^ or by determining transformations of features that lead to maximal enrichment of groups of related samples^89^. These strategies can highlight patterns that are not discriminated by regular metrics and that usually require more computational power to be calculated.

The performance of distance and similarity metrics relies on the quality of selected features (described in 'Feature selection'). High-dimensional feature profiles are often prone to the drawback of dimensionality, which consists of a decreasing ability of metrics to discern differences between vectors when the dimensionality increases. Dimensionality reduction can mitigate this effect (described in 'Linear transformations'). However, the choice of the metric can also be crucial, because good metrics better exploit the structure of the available features.

A comparison of metrics on one particular imaging data set has demonstrated that rank correlations (Spearman's and Kendall's) perform best for multiple untransformed feature vectors, whereas Euclidean and Manhattan distances are best for calculating *z*-prime factor values between positive and negative controls^78^. A comparison of metrics in gene expression data sets has suggested that Pearson's correlation performs best when features are ratios, whereas Euclidean distance is best on other distributions^105^.

The consensus from our laboratories is that selecting an optimal metric is probably specific to feature-space dimensionality and distributions that result from prior steps in the pipeline. For a typical pipeline, Pearson's correlation generally appears to be a good choice. Notably, indexes measuring clustering quality^106^, for example the Davies–Bouldin Index, silhouette statistic, and receiver operating characteristic–area under the curve can aid in metric choice^78,98^.

**Concentration-effect handling.** In experiments involving chemical perturbations, multiple concentrations are usually tested. Generally, researchers are interested in identifying phenotypic similarities among compounds even if those similarities occur at different doses. The following strategies are used to compute dose-independent similarity metrics:

*Titration-invariant similarity score*. First, the titration series of a compound is built by computing the similarity score between each dose and negative controls. Then, the set of scores is sorted by increasing dose and is split into subseries by using a window of certain size (for instance, windows of three doses). Two compounds are compared by computing the correlation between their subwindows, and only the maximum value is retained^83^.

*Maximum correlation*. For a set of *n* doses for each compound, the NxN correlation matrix is computed between all pairs of concentrations, and the maximum value is used as the dose-independent similarity score^72^.

The use of the maximum correlation is practical when a small number of concentrations are being tested. Depending on the experimental design, multiple concentrations can be treated differently. For instance, doses that do not yield a profile distinct from those of negative controls can be omitted, and the remaining doses can be combined to yield a single profile for the compound. Alternatively, if all concentrations are expected to have a phenotype, an entire compound can be left out of the analysis when its doses do not cluster together consistently^107^. In addition, high doses can be removed if they are observed to be too toxic according to certain criteria, such as a minimum cell count^102,107^.

### Step 7: assay quality assessment

Assessing quality for morphological profiling assays can be challenging: basing the assessment on a few positive controls is not reassuring, but there are rarely a large number of controls available, nor are there other sources of ground truth. Every measured profile combines a mixture of the signal relating to the perturbation together with unintended effects such as batch effects and biological noise. Tuning the sample-preparation technique, choosing cell lines or incubation times, and choosing among alternatives within the computational pipeline all benefit from use of a quantitative indicator of whether the assay is better or worse as a result of particular design choices. Options include:

*Comparison to ground truth*. If the expected similarities between pairs of biological treatments are known, they can be used to validate predicted values. For instance, different concentrations of the same compound are expected to cluster together, and computed similarities should reflect that clustering. Similarly, if a subset of biological treatments is known to fall into particular classes, classification accuracy can be an appropriate metric^77^. However, it is challenging to obtain ground-truth annotations at a large scale. To our knowledge, the only publicly available image data set with a large number of class annotations is for human MCF7 breast cancer cells (in this case, various classes of compound 'mechanisms of action')^108^. Importantly, for proper evaluation of this data set, one complete compound set, including all concentrations, should be left out of training. A common mistake is to leave out a single dose of a single compound, inappropriately leaving the remaining doses of the same compound available to the classifier for training. Additional benchmarks beyond this data set are greatly needed.

*Replicate reproducibility*. This is typically measured as the similarity among the profiles of replicate pairs of the same biological treatment, which should be significantly higher than the similarity to profiles of other experimental conditions (controls and/or other biological treatments). This procedure requires at least two replicates of the experiment, a condition usually met for modern morphological profiling experiments. To assess significance, similarity scores are compared with a suitable null distribution. A null distribution is usually built with pairs of samples that are not expected to be highly correlated, and it mainly depends on the hypothesis being tested. For instance, the use of all pairs of biological treatments can provide a diverse null distribution for measuring replicate correlation, and a null formed by random pairs of control samples can be compared against controls grouped by well location to reveal position effects. A *P* value can be computed nonparametrically by evaluating the probability of random pairs having greater similarity than a particular replicate pair.

*Effect size*. The difference between positive and negative controls, also known as the effect size, can be used as a measure of quality. This measure can be computed with a wide variety of statistical formulations, including univariate and multivariate methods, and also by assuming parametric and nonparametric models^109,110^. The disadvantage of this approach is that maximizing effect size alone may cause a bias toward detecting only those phenotypes that distinguish the control while ignoring other phenotypes.

*Exploratory approaches*. Several methods have not been tested but might prove useful. Clustering can be used to ascertain the overall structure of relationships among samples in the experiment: a pipeline that produces substructures or many distinct clusters is likely to be preferable over one in which the distances between all pairs of samples are similar. The cumulative variance of the principal components is a metric not yet applied to morphological profiling experiments. Highly diverse signals from different biological treatments should require more components to explain a predefined fraction of variance (for example, 99%).

Currently, replicate reproducibility is the most commonly used method, given that ground truth is rarely available. Specifically, methods are often optimized to maximize the percentage of replicates that are reproducible relative to a null (under suitable cross validation). Using a null comprising pairwise correlations between different treatments is safer than using a null comprising correlations between treatments and negative controls; in the latter case, it is possible to optimize the assay to distinguish samples from negative controls while diminishing important differences among samples.

### Step 8: downstream analysis

Downstream analysis is the process of interpreting and validating patterns in the morphological profiles. The most important readouts are the similarities and relationships among the experimental conditions tested. Visualization of the relationships and the use of machine learning can help to uncover biologically meaningful structures and connections among various treated samples. Most laboratories use a combination of these strategies; generally, unsupervised clustering is a good starting point for exploring the data. From there, the goals of the study strongly influence the combination of approaches used.

**Clustering.** Finding clusters is one of the most effective ways of extracting meaningful relationships from morphological profiles. Clustering algorithms can be used for identifying new associations among treatments as well as validating known connections and ruling out batch effects. There are several ways of clustering a data set. Hierarchical clustering, the most widely adopted strategy, is used to identify groups of highly correlated experimental conditions^87^ and to identify treatments with unexpected positive or negative connections^99^. Although it is not discussed in detail here, examining relationships among features rather than among samples can yield useful biological insights: for example, the amount of mitochondrial material in cells is generally proportional to cell size, thus revealing stereotyped control of these parameters, but certain chemical perturbants can disrupt this relationship^111^.

Hierarchical clustering is computed by using a similarity matrix that contains the similarity values for all pairs of samples (described in 'Measuring profile similarity'). This similarity matrix can be visualized as a heat map to reveal patterns in the data for several or up to hundreds of samples. The heat maps' rows and columns are typically sorted by using the hierarchical structure discovered by the clustering algorithm. This hierarchical structure is known as a dendrogram, which links samples together according to their proximity in the feature space, and is usually visualized together with the heat map to highlight negative and positive correlations in the data (Fig. 6a). Bootstrapping has been used to evaluate the statistical significance of the results obtained with hierarchical clustering, as well as other probabilistic algorithms used in the analysis of single-cell populations^32^. Resampling methods can generally be used to estimate variance, error bars, or other statistical properties of the data and can aid in making more accurate predictions and interpretations.

**Visualization of high-dimensional data.** Visualizations are useful to reveal the distribution and grouping of high-dimensional data points by using a 2D (and sometimes 3D) map layout that approximates their positions in the feature space. The relationships among points are implicitly encoded in how close together or far apart they are in the visualization. This method can be used to study cell heterogeneity by using single-cell data points, or sample relations by using aggregated profiles. Single-cell data are usually downsampled for practical reasons: to decrease data size and identify rare cell types^112,113^. The following are the most common approaches for data visualization:

*Data projections*. A projection of the data matrix is displayed in a 2D (or 3D) scatter plot that approximates the geometry of the original point cloud. The most common methods include PCA (Fig. 6b), Isomap^114^, *t-*distributed stochastic neighbor embedding (tSNE)^115^ (Fig. 6c), and viSNE^116^.

*Hierarchical visualizations*. Plots are used to find structures in the data and reveal relationships between samples (Fig. 6d,e). The most commonly used choices are spanning-tree progression analysis of density-normalized events (SPADE)^113,117^ and minimum spanning trees^118^, which allow for relationships among hierarchical groups of single cells or samples to be identified by using branches that may represent phenotypes or treatments.

In many cases, data points in a visualization are colored on the basis of positive controls or otherwise known labels in the data, a common practice in analysis of single-cell flow cytometry data^116,119,120^. The color code can also illustrate other information in the data set, such as cell phenotypes, compound doses, values of measured features, or treatment conditions (Fig. 6e). Visualizations can be more effective if they are interactive, thereby allowing researchers to create and test hypotheses *ad hoc*. Software packages such as Shiny, GGobi, iPlots in R, Bokeh in Python, and D3.js in Javascript provide interactive plotting capacities, most of which can also be deployed in server-client environments for dissemination to the public.

**Classification.** Classification rules can be useful for transferring labels from annotated samples to unknown data points, for example, classifying the mechanism of action of new compounds in a chemical library. As such, classification strategies require prior knowledge in the form of annotations for at least some of the data points in the collection. Given examples of data points that belong to different classes of interest, supervised classification algorithms learn a rule that computes the probability of each unknown data point falling into one of the classes.

It is relatively uncommon to have a large number of annotated samples in morphological profiling, because most experiments are designed to be exploratory. However, when this information is available, a classification strategy can provide informative insights into the treatments. The most commonly used classification rule in morphological profiling experiments is the nearest-neighbors algorithm, which finds the closest data points in the collection of annotated samples and recommends a label for the new sample. For instance, this algorithm has been used for classifying the mechanism of action in a compound library^77^. Other supervised prediction models can also be used to learn relations between morphological features and biological activity assays, such as Bayesian matrix factorization, neural networks, and random forests^121^.

The classification performance is validated in a holdout test using precision, recall, and accuracy measures. It is absolutely critical for confidence in these metrics that the holdout test set not overlap with any data points in the training set. The most recommended practice is to use samples treated in a different experimental batch to create the holdout test set (other ground-truth recommendations are described in 'Assay quality assessment').

### Sharing

Both authors and the scientific community benefit from sharing code and data^122^. Numerous tools currently exist that address the steps outlined in this paper (Box 1); these tools can be useful both for beginners to experiment with and learn from and for experts to integrate into pipelines and build upon. Although data must be kept confidential for sensitive patient material, intellectual-property concerns are generally not the major issue with sharing; the primary hurdle in the process is usually the often substantial time and effort required of the authors. We do not consider code or data labeled 'available upon request' to qualify as being openly shared, given the poor efficacy statistics^123,124^. We therefore recommend the following options to make code and data available publicly online.

**Code sharing.** Options for sharing code include:

*Step-by-step narrative*. For software with only a graphical user interface, a detailed walkthrough of each step of the workflow can be provided; however, this option is suboptimal.

*Online code repository*. The code should preferably be publicly hosted rather than being provided on a university website or as journal supplemental files. The options range from repositories such as Github and BitBucket to tools such as Jupyter notebooks and knitr documents^125^, which allow for reproducible reports containing code, documentation, and figures to be shared within a single document.

*Packaging*. Researchers can capture and share the computational environment used to create the results, such as providing virtual machines or Docker containers. Doing so ensures that all code, dependencies and data are available in a single container^126,127^, which is convenient for the user and also protects against changes in software libraries and dependencies.

**Data sharing.** In image-based cell profiling, publicly available data are valuable not only for reproducing results but also for identifying completely new biological findings. Options include:

*Sharing processed data only*. Sharing only processed data (for example, extracted features) has been common, often through supplemental data files available via the journal or via a general-purpose data repository such as Dryad (http://datadryad.org/).

*Sharing images and data online*. Few raw-image sets have been made available online, primarily because of the large size of the image data (tens of gigabytes for each 384-well plate) and therefore the high cost of maintaining the data on public servers. However, recent initiatives are decreasing this cost for authors, including the Image Data Resource (IDR; https://idr-demo.openmicroscopy.org/)^128^, which accepts cellular images at the scale of high-throughput image profiling experiments. Generally, smaller sets of annotated images for testing image analysis methods are available in the Broad Bioimage Benchmark Collection (https://data.broadinstitute.org/bbbc/)^108^ and the Cell Image Library (http://www.cellimagelibrary.org/). Some resources, such as IDR, support using an ontology for describing phenotypes^129^. Before these public resources became available, some laboratories provided the data through their institutional servers^13,32,52,89,103,130,131^. Tools such as OMERO^132^ and openBIS^133^ have been used to create project-specific portals for easy online data exploration^32,52,130^, but bulk download of very large data sets can remain challenging.

We strongly encourage sharing of both data and images online, given how rapidly feature-extraction methods are changing, particularly via deep-learning methods (described in 'Alternate workflows').

Box 1: SOFTWARE TOOLS A large range of software tools and libraries currently exist that seek to address the steps outlined in this paper. For each step, the alternatives are usually several software packages or programming languages that require either parameterization or coding.

Tools for image-analysis software have been previously reviewed^150^, and the variety in functionalities and platforms can fit a diverse range of workflows. Some of the open-source alternatives include CellProfiler^24^ and EBImage^35^, whereas Columbus and MetaXpress are commercial solutions.

After collection of features or measurements with image-analysis software, the next steps in the workflow may require a combination of tools and programming languages. Statistical packages such as R have proven to be very useful for single-cell data analysis, including *cytominer*, which is specific to morphological profiling. Other programming languages such as Python, Matlab and shell scripts can be used to process data with specific algorithms, including machine learning, data transformation, or simple data filtering and selection.

Each step may require specialized methods or may be solved with off-the-shelf implementations. The field is constantly changing, and the next breakthroughs in theory and practice may require new tools not yet available. In either case, the practice of sharing code is highly valued, to ensure rapid implementation of techniques, optimization of pipelines, and reproducibility of the results by others.

### Alternate workflows

The data-processing workflow and recommendations presented in this paper have evolved as a result of years of efforts in different laboratories. They have been robustly used in various studies and have proven to be successful in making biological discoveries^8,9^. However, the field is eager to adapt as the computer-vision and machine-learning communities make progress in designing new algorithms for processing image data. Some of our laboratories are already exploring alternate workflows, such as those described below.

**Segmentation-free classical-feature extraction.** Instead of identifying single cells that are measured and characterized, this strategy computes classical features from whole field-of-view images or from discrete tiles within images. Examples of these include PhenoRipper^134,135^ and WND-Charm/CP-CHARM^136,137,138^.

**Deep-learning feature extraction.** Deep learning techniques have recently and dramatically come to dominate the state-of-the-art performance in various computer vision tasks^139^. The most relevant model for image analysis is currently the convolutional neural network (CNN), which learns to extract useful features directly from raw pixel data by using multiple nonlinear transformations, in contrast to the classical features described in 'Feature extraction'. This model has been used for segmentation and classification of biomedical images^140,141^, for phenotype discovery in single-cell images from imaging flow cytometry^142^, and more recently for deep-learning approaches for morphological profiling: morphological profiling^143,144^. The following are the most relevant deep-learning approaches for morphological profiling:

*Learning features from raw pixels*. This approach has been used for problems in which phenotypes of interest are predefined, and a set of categorized examples is needed to train the network. This approach has been successfully used for protein-localization problems^145,146,147^ and mechanism-of-action prediction^144^. Input images can be single cells^146,147^ or full fields of view^144,145^.

*Transferring learned features from other domains*. Using a CNN trained on a large data set for other tasks different from the original is known as transfer learning. CNNs pretrained with natural images have been evaluated as feature extractors for full image profiling of compounds; its accuracy matches the results of classical features without requiring segmentation or training^143^. The preprocessing steps described in 'Field-of-view quality control' and 'Field-of-view illumination correction' are still likely to be necessary for obtaining improved results. If there are few annotations available for phenotype-classification tasks, transfer learning can also be used to improve performance^146^.

*Learning transformations of classical features*. feature transformations similar to those described in 'Linear transformations' can be obtained with a technique known as the autoencoder. Deep autoencoders have been evaluated for high-content morphology data, thus suggesting that they may potentially have better performance for downstream analysis according to homogeneity of clusters^148^. Another study has evaluated deep autoencoders for profiling and has also obtained competitive performance^149^.

Using full images results in a loss of single-cell resolution but offers several advantages: the avoidance of the segmentation step eliminates the sometimes tedious manual tuning of segmentation and feature extraction algorithms, saves computation time, avoids segmentation errors, and may better capture visual patterns resulting from multiple cells. Using single-cell images explicitly captures heterogeneity and may offer improved accuracy with less training.

Although segmentation-free classical-feature extraction can be helpful for quality control, we generally consider it to be incapable of accomplishing most profiling tasks. Deep-learning techniques, although not yet proven to be more powerful than the standard workflow, are nonetheless very promising. We are actively pursuing optimized workflows based on deep learning and are gaining an understanding of how these techniques can be adapted for improving the computation and interpretation of useful image features.

We caution that it is possible to obtain excellent results on a ground-truth data set with a method that fails in realistic-use cases. This phenomenon may be especially true for machine-learning-based methods with millions of internal parameters and again reinforces the need for new and disparate sets of ground-truth data in the field.

## Conclusions

It is an exciting time for the field of image-based cell profiling, as methods are rapidly evolving and applications leading to major biological discoveries are beginning to be published. We see the collection and sharing of large biologically interesting image sets, the organizing of benchmark ground-truth data sets, and the testing of new methods to be the major areas in which effort is currently most needed.

In future work, as a community, we aim to build shared codebases, namely toolboxes of algorithms in R and Python. The beginnings of this effort can be found online (https://github.com/CellProfiler/cytominer/), and we welcome additional contributors as well as participants in the cytomining hackathon, which will be held annually. A shared codebase will facilitate the development and dissemination of novel methods and the comparison of alternative methods, particularly as additional ground-truth data become publicly available.

## Data availability

This work did not analyze new data. The plots and figures presented in the manuscript were obtained by processing the BBBC021 image collection, which is publicly available in https://data.broadinstitute.org/bbbc/BBBC021/.
